# Finding a Missing Gene: *EFG1* Regulates Morphogenesis in *Candida tropicalis*

**DOI:** 10.1534/g3.115.017566

**Published:** 2015-03-09

**Authors:** Eugenio Mancera, Allison M. Porman, Christina A. Cuomo, Richard J. Bennett, Alexander D. Johnson

**Affiliations:** * Department of Microbiology and Immunology and; † Department of Biochemistry and Biophysics, University of California San Francisco, San Francisco, California 94158; ‡ Department of Microbiology and Immunology, Brown University, Providence, Rhode Island 02912; § Broad Institute of MIT and Harvard, Cambridge, Massachusetts 02142

**Keywords:** *Candida* morphogenesis, biofilm, filamentation, phenotypic switch, transcriptional regulation

## Abstract

Fungi from the genus *Candida* are common members of the human microbiota; however, they are also important opportunistic pathogens in immunocompromised hosts. Several morphological transitions have been linked to the ability of these fungi to occupy the different ecological niches in the human body. The transcription factor Efg1 from the APSES family plays a central role in the transcription circuits underlying several of these morphological changes. In *Candida albicans*, for example, Efg1 is a central regulator of filamentation, biofilm formation, and white-opaque switching, processes associated with survival in the human host. Orthologs of Efg1 are present throughout the *Candida* clade but, surprisingly, the genome sequence of *Candida tropicalis* failed to uncover a gene coding for Efg1. One possibility was that the paralog of Efg1, Efh1, had assumed the function of Efg1 in *C. tropicalis*. However, we show that this gene has only a minor role in the morphological transitions mentioned above. Instead, we report here that *C. tropicalis* does have an ortholog of the *EFG1* gene found in other *Candida* species. The gene is located in a different genomic position than *EFG1* in *C. albicans*, in a region that contains a gap in the current genome assembly of *C. tropicalis*. We show that the newly identified *C. tropicalis EFG1* gene regulates filamentation, biofilm formation, and white-opaque switching. Our results highlight the conserved role of Efg1 in controlling morphogenesis in *Candida* species and remind us that published genome sequences are drafts that require continuous curation and careful scrutiny.

Several species of the *Candida* genus belong to a monophyletic clade of ascomycetous fungi that translate the CTG codon as serine instead of leucine (Butler *et al.* 2009). Members of this CTG clade include species that are commensals of the human microbiota with no known environmental reservoirs outside of animals. However, these commensals can also cause mucosal disease in healthy individuals as well as systemic infections in immunocompromised hosts (Calderone 2002). Members of the CTG clade, therefore, are important fungal human pathogens, both in terms of their prevalence and their mortality rate.

Although they are most commonly encountered in the yeast form (unicellular spherical cells), most CTG species are able to undergo a variety of changes in cell and colony morphology. The best studied of these is the ability to switch between the yeast and hyphal (filamentous) forms, a transition that is closely linked to pathogenesis and biofilm formation (Sudbery 2011). Biofilms are communities of cells associated with a biologic or inert surface. In the case of *Candida* species, these communities are typically composed of several layers of yeast cells and hyphae that are embedded within an extracellular matrix (Finkel and Mitchell 2011; Nobile *et al.* 2012). Other morphological transitions include the phenomenon of heritable phenotypic switching. In *C. albicans*, this includes the white-opaque switch, in which cells transition between two different cell types that generate either shiny and dome-shaped colonies (white) or dull and flat colonies (opaque) (Slutsky *et al.* 1987; Lohse and Johnson 2009). White and opaque cells are morphologically distinct, have different metabolic preferences, and differ in their ability to mate (Slutsky *et al.* 1987; Lan *et al.* 2002; Miller and Johnson 2002). Several lines of evidence suggest that morphological transitions allow *C. albicans* to adapt to different ecological niches in the human host (Kvaal *et al.* 1997; Lohse and Johnson 2009; Pande *et al.* 2013; Tao *et al.* 2014). For example, filamentous cells are more effective at invading epithelia, whereas biofilms often form on indwelling medical devices and confer tolerance to antifungal drugs (Cutler 1991; Donlan 2001; Donlan and Costerton 2002; Kojic and Darouiche 2004; Sudbery 2011). White and opaque cells are also known to differ in their ability to colonize different anatomical locations and are distinct in terms of their virulence (Kvaal *et al.* 1997; Lachke *et al.* 2003; Lohse and Johnson 2009). Therefore, elucidating the molecular mechanisms underlying phenotypic changes will help determine how these fungi are able to colonize and infect multiple niches in the human body.

At the molecular level, the morphological changes described above have been extensively investigated in *C. albicans*. Filamentation, biofilm formation, and white-opaque switching are regulated by specific sets of transcription factors that control the expression of large numbers of target genes (Kadosh and Johnson 2005; Nobile *et al.* 2012; Hernday *et al.* 2013). Interestingly, the transcription factor Efg1 is unique in being common to all three transcription circuits. This transcription factor was originally identified as a regulator of hyphae formation and a member of the fungal-specific APSES family of DNA binding proteins (Stoldt *et al.* 1997). In *C. albicans* it acts as an activator or repressor of hyphae formation depending on the environmental conditions (Lo *et al.* 1997; Stoldt *et al.* 1997; Tebarth *et al.* 2003). Efg1 is also one of the six core transcription factors that control biofilm formation in *C. albicans*. Its deletion has the strongest effect on biofilm formation *in vitro* among the six core biofilm transcription factors (Nobile *et al.* 2012). In white-opaque switching, Efg1 is a critical transcription factor for formation of the white phenotypic state (Sonneborn *et al.* 1999; Srikantha *et al.* 2000; Zordan *et al.* 2007). Efg1 binds to the regulatory sequences of the five other transcription factors of the circuit and represses the expression of the master regulator of the opaque state, Wor1 (Zordan *et al.* 2007; Hernday *et al.* 2013). In addition to its role in these phenotypes and possibly as a consequence, Efg1 plays an important role in mediating colonization of the gastrointestinal tract (Pierce and Kumamoto 2012; Pande *et al.* 2013; Hirakawa *et al.* 2014). Together, these studies establish the central role of Efg1 in regulating multiple facets of *C. albicans* biology, including both commensalism and pathogenicity.

Relatively little is known about the role of *EFG1* orthologs in other CTG species. In *Candida parapsilosis*, Efg1 is involved in biofilm formation and also regulates a colony morphology switch, although the morphologies appear distinct from *C. albicans* white and opaque states (Connolly *et al.* 2013). Interestingly, the role of Efg1 as a regulator of morphological change appears to be conserved even in species outside the CTG clade. For example, the ortholog of Efg1 in the model yeast *Saccharomyces cerevisiae*, Sok2, controls pseudohyphal growth (Pan and Heitman 2000), whereas in the filamentous ascomycetes *Neurospora crassa* and *Aspergillus nidulans*, the corresponding orthologs regulate ascospore maturation and conidiosphore development, respectively (Aramayo *et al.* 1996; Dutton *et al.* 1997).

Given the central role that *EFG1* plays in adaptation of *C. albicans* to the human host and in morphological transitions across multiple fungal species, it was surprising that the genome sequence of *C. tropicalis* appeared to be missing an ortholog of this gene (Butler *et al.* 2009; Maguire *et al.* 2013). *C. tropicalis* is a CTG species related to *C. albicans* [approximately 55 million years since their last common ancestor (Mishra *et al.* 2007)] and is a common commensal and pathogen of humans (Butler *et al.* 2009). *C. tropicalis* is also known to undergo the yeast-to-hyphae switch, to form biofilms, and to exhibit white-opaque switching (Porman *et al.* 2011; Xie *et al.* 2012; Porman *et al.* 2013). Recent experiments have revealed that the *C. tropicalis* white-opaque switch involves a third stable state, termed the "intermediate" state, and therefore exhibits tristable rather than bistable switching (A. M. Porman, M. Anderson, N. Wang, E. Mancera, and R. J. Bennett, unpublished data). Among all CTG species whose genomes have been sequenced, *C. tropicalis* is the only one apparently missing an ortholog of *EFG1* (Maguire *et al.* 2013).

One possibility to explain the absence of *EFG1* in *C. tropicalis* is that its functional role has been assumed by the APSES paralog *EFH1*, but we show here that *EFH1* plays only a minor role, if at all, in the morphological transitions mentioned above. Instead, we report here that the genome of *C. tropicalis* does contain an ortholog of *EFG1*, but it was not incorporated in the final assembly of the genome sequence. We first uncovered the gene by PCR amplification from genomic DNA using primers designed on *EFG1* orthologs from related CTG species. We subsequently determined the sequence and genomic location of *C. tropicalis EFG1*. The *C. tropicalis* gene lies in a different genomic location than that of its ortholog in *C. albicans* and yet clearly represents a true *EFG1* ortholog. Deletion of both alleles of the gene revealed that it is critical for filamentation, biofilm formation, and white-opaque switching in *C. tropicalis*, similar to its role in *C. albicans*. Overall, our results stress the conserved role of *EFG1* in regulating physical transitions in fungi and provide a cautionary note for the analysis of published genome sequences.

## Materials and Methods

### Strain/plasmid construction

Transformations were performed as previously described for *C. tropicalis* (Porman *et al.* 2011). Nutritional gene deletions were constructed using the *SAT1* flipper strategy (Reuss *et al.* 2004) in two different *C. tropicalis* genetic backgrounds: MYA3404 (the strain used by the genome sequencing project) and AM2005/0093. Plasmids to delete *HIS1*, *LEU2*, and *ARG4* were made as described (Noble and Johnson 2005). For the deletion of *EFH1* and *EFG1,* the 5′ and 3′ ∼300-bp regions flanking the ORFs were PCR-amplified. *HIS1*, *LEU2*, and *ARG4* auxotrophic markers were PCR-amplified from plasmids pSN52, pSN40, and pSN69, respectively. Fusion PCR was then performed to fuse 5′ and 3′ flanks to nutritional markers. This PCR product was transformed into auxotrophic strains lacking *HIS1* and *LEU2* or *HIS1* and *ARG4* (Supporting Information, Table S2) and the process repeated to delete the second copy of the ORF. When using strains that were only leu minus, the first allele was deleted using the *LEU2* marker from plasmid pEM002 and the second allele using the HYG marker from plasmid pYM70 (Basso *et al.* 2010). PCR was used to confirm correct genomic integration of the markers and homozygous deletion of the genes. Two independent isolates were generated for each deleted gene in each genetic background (Table S2).

### Cloning *EFG1* from *C. tropicalis*

The amplification of the first fragment of *EFG1* was performed by PCR using degenerate primers designed against the portion of the *EFG1* sequence that is most conserved from *C. albicans* to *C. parapsilosis* (Table S1, primers EMO348 and EMO352). *C. tropicalis* genomic DNA from the *EFH1* deletion mutant was used as template and annealing temperatures of between 40° and 50° were used. Based on the sequence of the amplified fragment and conserved regions further out on the *C. albicans* gene, more primer pairs were designed to further extend the amplified region by PCR (Table S1).

For the final cloning of the gene, two flanking ∼300-bp segments in the center of the *EFG1* sequence identified above were PCR-amplified from genomic DNA to be used as homology for integration. These two regions were PCR-fused to the edges of a segment containing the *SAT1* marker, the CamR marker, and an *E. coli* origin of replication that was PCR-amplified from plasmid pSFS2A (Reuss *et al.* 2004). The resulting cassette (5′ *EFG1* homology—drug markers and origin of replication—3′ *EFG1* homology) was transformed into *C. tropicalis* cells as previously described. Transformants with the correct insertion at the center of the endogenous *EFG1* sequence were selected on YEPD plates containing 400 μg/ml of nourseothricin and verified by colony PCR. Genomic DNA from the transformants was extracted, digested with *Xho*I and *Nhe*I separately, circularized by ligation, and electroporated into *E. coli* XL1-Blue Electroporation-Competent Cells (Agilent Technologies) following the manufacturer’s protocol. Clones containing the *EFG1* gene with the *SAT1*- CamR cassette were selected on LB plates containing 34 μg/ml chloramphenicol. Sequencing of the clones was performed by primer walking.

### Characterization of hyphae formation

Similarly to what has been previously described (Lackey *et al.* 2013), cells from a YEPD, 30°, overnight culture, were washed twice with water and used to inoculate SD medium supplemented with 0.75% glucose and 50% fetal bovine serum at an OD_600_ of 1.5. This culture was grown with shaking at 37°. Samples were taken at the time of inoculation (0-hr time point) and at 2, 4, and 22 hr after inoculation and were visualized by differential interference contrast (DIC) microscopy.

### Characterization of biofilm formation

Biofilm formation was assayed by confocal scanning laser microscopy and by determining biomass dry weight as previously described (Nobile *et al.* 2012). Briefly, overnight YEPD cultures grown at 30° were used to inoculate silicon squares pretreated with adult bovine serum at an OD_600_ of 0.5. Cells were left to adhere to the silicon square in Spider medium without mannitol and with 1% glucose at 37° and shaking at 200 rpm for 90 min. The squares were then washed with PBS and transferred to fresh Spider 1% glucose medium to be incubated for 48 hr at 37° and shaking at 200 rpm. Biofilms formed on the squares were stained for 1 hr with 50 mg/ml of concanavalin A Alexa Fluor 594 conjugate and visualized on a confocal microscope using a 40×/0.80W Nikon objective. For the determination of biomass dry weights, biofilms were grown in the same way, but directly on the bottom of six-well polystyrene plates. After 48 hr of growth at 37°, the medium was aspirated, biofilms were scraped from the bottom of each well, and they were dried on top of preweighed 0.8-μm nitrocellulose filter papers. Five replicate biofilms grown in separate wells were used for each strain.

### Phenotypic switching assays on *C. tropicalis* mutant strains

Strains were grown overnight at 30° in Spider medium. Cells were diluted in water and plated onto Spider medium at a concentration of ∼100 colonies per plate. Plates were incubated for 7–10 days at 30° and colonies were examined for sectors. Cellular morphology was assessed by DIC microscopy of cells from the resulting colonies. Starting cells for each strain were in the corresponding default state: white, intermediate, and opaque for the wild-type, *efg1Δ/EFG1*, and *efg1Δ/efg1Δ* strains, respectively. For the heterozygous and homozygous mutants, we were not able to obtain stable populations in the white state.

## Results

### The role of *EFH1* in *C. tropicalis* morphogenesis

Given the apparent absence of an ortholog of *EFG1* in *C. tropicalis*, it could be hypothesized that the role of *EFG1* had been assumed by its APSES paralog, *EFH1*. To test this, we generated deletion mutants of this gene in *C. tropicalis* and evaluated filamentation, biofilm, and white-opaque switching phenotypes. Filamentation was assayed by growth at 37° in SD medium supplemented with 0.75% glucose and 50% fetal bovine serum. This medium has been previously reported to be optimal for the induction of *C. tropicalis* filamentation (Lackey *et al.* 2013). Hyphae formation was monitored at 2, 4, and 22 hr of growth in filamentation-inducing medium. No filamentation defect was observed in the *efh1*Δ/*efh1*Δ mutant compared to the wild-type strain at any of the examined time points. Biofilms were generated by growth of *C. tropicalis* cells on silicon or polystyrene surfaces for 48 hr (see *Materials and Methods*). Similar to what has been observed in *C. albicans* (Nobile *et al.* 2012), biofilms formed by *C. tropicalis efh1Δ/efh1Δ* deletion mutants were indistinguishable from those formed by the wild-type strain when grown on a silicon surface and visualized by confocal microscopy. When grown on polystyrene plates, *efh1Δ/efh1Δ* biofilms did appear different from the wild-type in terms of their structure but showed similar overall thickness and biomass (10.87 mg ± 0.5 in *efh1Δ/efh1Δ vs.* 11.62 mg ± 0.4 in WT). The deletion of *EFH1* in *C. tropicalis* also had no significant effect on the frequency with which this species switches from the white to the opaque state (A. M. Porman, M. Anderson, N. Wang, E. Mancera, and R. J. Bennett, unpublished data). This is in agreement with previous reports showing that the expression levels of *EFH1* in *C. tropicalis* are similar between white and opaque cells (Porman *et al.* 2011). Taken together, these results indicate that the role of *EFG1* in *C. tropicalis* has not simply been assumed by its paralog *EFH1*.

### Identification of an ortholog of *EFG1* in *C. tropicalis*

To test whether *C. tropicalis* has an *EFG1* gene that was missed by sequencing analysis, we performed PCR on *C. tropicalis* genomic DNA using degenerate primers designed to anneal to conserved regions of *EFG1* orthologs from the CTG clade (Table S1). We used low annealing temperatures to allow for mismatches between primers and template, and we used genomic DNA from the *efh1Δ/efh1Δ* mutant to avoid PCR amplification of the paralog gene. This approach allowed us to PCR amplify a ∼260-bp sequence that was sequenced and shown to be more similar to *C. albicans EFG1* than to *C. tropicalis EFH1*. Additional rounds of PCR using primers that anneal to the amplified region or to the *C. albicans EFG1* gene allowed us to extend the sequenced region to 900 bp. This 900-bp region did not match any sequences in the assembled *C. tropicalis* genomic sequence that would allow placement of the gene in the genome.

**Figure 1:**
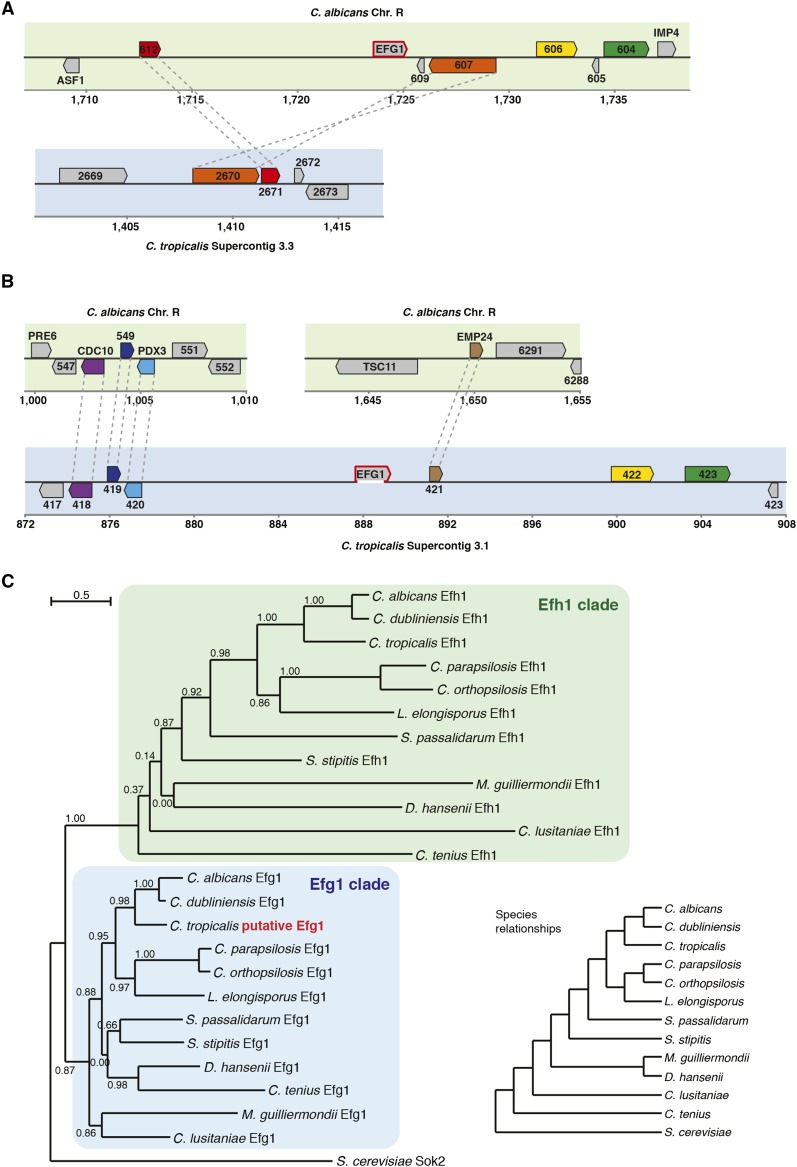
Genomic and phylogenetic position of *EFG1* in *C. tropicalis*. (A) Schematic depiction of the genomic location of *EFG1* in *C. albicans* and the corresponding genomic location in *C. tropicalis*. (B) As (A), but for *C. tropicalis EFG1* and the corresponding location in *C. albicans*. In (A and B) the upper panel depicts *C. albicans* chromosomes (green background) and the lower panel depicts *C. tropicalis* chromosomes (blue background). Coordinates are in kb. One-to-one orthologs defined by CGOB are shown using the same color and are connected by dashed gray lines if in the same panel. The prefixes “orf19.” and “CTRG_” for *C. albicans* and *C. tropicalis* ORF names, respectively, were omitted. The white segment at the bottom of the *C. tropicalis EFG1* gene depicts the location of the sequence gap. (C) Gene phylogeny of the *EFG1* and *EFH1* genes in the CTG clade and *S. cerevisiae*. Ortholog sequences were obtained from CGOB (Maguire *et al.* 2013), aligned using MUSCLE, and a phylogenetic tree was generated using PhyML in Seaview (Gouy *et al.* 2010). Branch support values are SH-like approximate likelihood ratios and the branch-length scale bar represents substitutions per site. Generating the tree by bootstrapping (100 trees) gave the same tree topology. The species relationships depicted in the cladogram (lower right) were obtained from (Maguire *et al.* 2013).

To identify the complete sequence of the *C. tropicalis EFG1* gene and to map its location in the genome, we used a targeting plasmid to integrate into the endogenous *EFG1* locus. The plasmid contained the CamR drug marker selectable in *Escherichia coli* and the *SAT1* marker selectable in *Candida* species. From this plasmid we generated a PCR cassette that contained the two selectable markers and an *E. coli* origin of replication, together with flanking arms with homology to the sequenced region of *C. tropicalis EFG1*. This cassette was transformed into *C. tropicalis* cells, where it inserted at the endogenous *EFG1* gene by homologous recombination. Genomic DNA from these transformants was fragmented, circularized by ligation, and transformed into *E. coli*. Clones containing the putative *C. tropicalis EFG1* gene were selected and sequencing of these plasmids revealed an ORF of 1701 bp, found to map to Supercontig 3.1 of the *C. tropicalis* genome sequence. Notably, the ORF spans a gap in the Supercontig beginning at 887,643 bp and ending at 888,943 bp. The ORF starts 13 bp upstream of the gap and ends 342 bp downstream of it (see Figure 1). Based on the neighboring genes, the genomic location of *EFG1* in *C. tropicalis* is different from that of *EFG1* in *C. albicans* (Figure 1). This is highlighted by the fact that the *C. tropicalis* orthologs of the genes neighboring *EFG1* in *C. albicans* are located on a different Supercontig (Figure 1).

### Sequence analysis of the *EFG1* ortholog in *C. tropicalis*

Translation of the *C. tropicalis EFG1* ORF results in a polypeptide of 567 amino acids that has 54% sequence identity and 60% sequence similarity to the *C. albicans* Efg1 protein. The two Efg1 proteins also represent the best reciprocal BLAST hit among the genes of the *C. tropicalis* and *C. albicans* genomes. As can be observed in Figure 1, the identified gene is at the precise position in the phylogenetic tree where it would be expected given the species evolutionary relationships. It groups with the Efg1 orthologs in the CTG clade, closest to *C. albicans* and *Candida dubliniensis*, and in a distinct part of the tree from the *EFH1* orthologs. *C. tropicalis* Efg1 has a single conserved domain between amino acids 190 and 296 that matches the helix-turn-helix DNA binding domain characteristic of the APSES transcription factors (Stoldt *et al.* 1997). The domain in the *C. tropicalis* gene is almost identical to the APSES domain of *C. albicans* Efg1, differing only by two amino acids (sequence identity of 98%). Thus, by several criteria, the *C. tropicalis EFG1* is an ortholog of the family of *EFG1* genes from related CTG species.

We were able to reconstruct the two alleles of the *C. tropicalis EFG1* gene by comparing differences between sequenced clones (Sequence accession numbers KP314278 and KP314279). The two alleles differ by seven nucleotides, five of which are nonsynonymous. None of the allelic differences lie within the APSES DNA binding domain of the protein. These are the two alleles present in the *C. tropicalis* strain MYA3404, which was the strain used for the genome sequencing project (Butler *et al.* 2009). Although we also used a strain of another genetic background to confirm our functional results (described below), all of the findings presented here are for the MYA3404 strain background.

The upstream intergenic region of *EFG1* in *C. tropicalis* is strikingly long (10,110 bp compared with an average intergenic region in the *C. tropicalis* genome of 902 bp (Butler *et al.* 2009) and, in this sense, resembles the control region of *C. albicans EFG1* (9,831 bp or 10,077 bp if the tRNA in the intergenic region is excluded). In *C. albicans* it is believed that the extended control region helps explain the complex regulation of *EFG1* expression (Lachke *et al.* 2003; Tebarth *et al.* 2003; Nobile *et al.* 2012; Hernday *et al.* 2013).

### *C. tropicalis EFG1* is involved in filamentation, biofilm formation, and white-opaque switching

**Figure 2:**
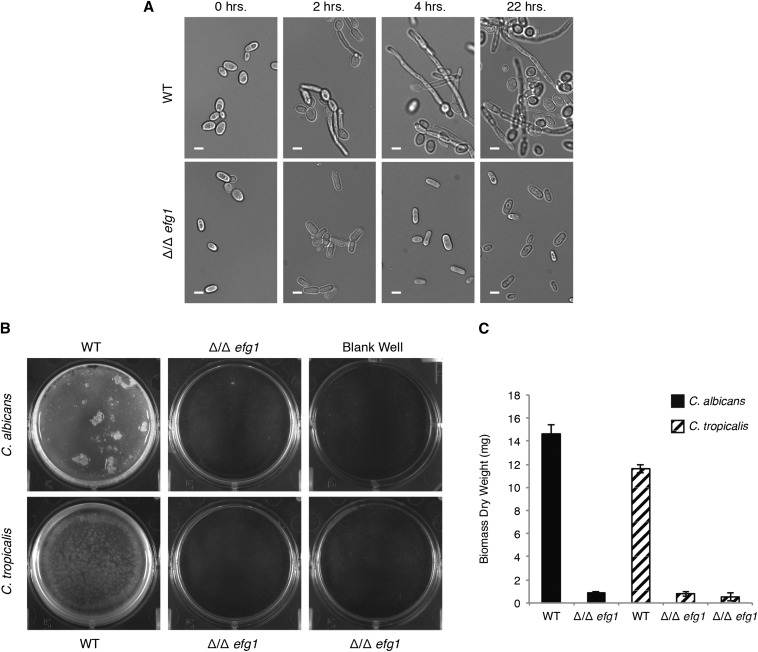
*EFG1* is involved in filamentation and biofilm formation in *C. tropicalis*. (A) Formation of filamentous cells by wild-type and *EFG1* deletion strains of *C. tropicalis* in the MYA3404 genetic background. Representative DIC microscopy images are shown for each time point. Scale bars are 5 μm. (B) Images of representative biofilms for *EFG1* deletion mutants in *C. albicans* and *C. trpicalis* (two independent isolates) grown in wells of six-well polystyrene plates. (C) Biomass dry weights of the same strains shown in (B), grown in the same conditions. Error bars represent the SD of five replicates.

We generated deletion mutants of *C. tropicalis EFG1* to characterize the role of the gene in morphogenesis (Table S2). Two independent isolates of the deletion mutant were tested to ensure that the phenotypes observed were not due to off-target effects. The deletion mutants were assayed for filamentation as described above for the *EFH1* deletion mutants. As can be seen in Figure 2, the deletion of *EFG1* in *C. tropicalis* had a strong effect on hyphae formation, with cells remaining in the yeast form even after 22 hr in the filamentation-inducing conditions. In contrast, cells from the wild-type strain showed hyphal projections even at the 2-hr time point. This phenotype is in agreement with the observed phenotype in *C. albicans* when *EFG1* is deleted (Lo *et al.* 1997).

To assess whether *EFG1* is involved in biofilm formation in *C. tropicalis*, we determined the ability of the deletion mutant to form biofilms on two different *in vitro* surfaces, silicon and polystyrene, both pretreated with bovine serum. Biofilm accumulation was determined by confocal microscopy and dry weight measurements. After testing a variety of media, we performed assays in Spider medium with mannitol replaced by 1% glucose, because this medium was optimal for *C. tropicalis* biofilm formation. The ability to form biofilms by *C. tropicalis* was severely impaired in the *efg1Δ/efg1Δ* mutant compared to the wild-type strain (Figure 2). Using confocal microscopy, biofilms formed by the *efg1Δ/efg1Δ* mutant were composed only of layers of yeast cells with very few filamentous cells. Also, the few filamentous cells that were present resembled pseudohyphae rather than true hyphae. In contrast, wild-type strains formed biofilms that are multilayered with a thick layer of hyphal cells on top of basal layers of yeast cells. The biofilm deficiency in the *efg1Δ/efg1Δ* mutant was also evident in the overall biomass of the biofilm (Figure 2). As shown in Figure 2, the biofilm defect observed in the *C. tropicalis* deletion mutant resembles the *C. albicans* phenotype when the orthologous gene is deleted.

**Figure 3:**
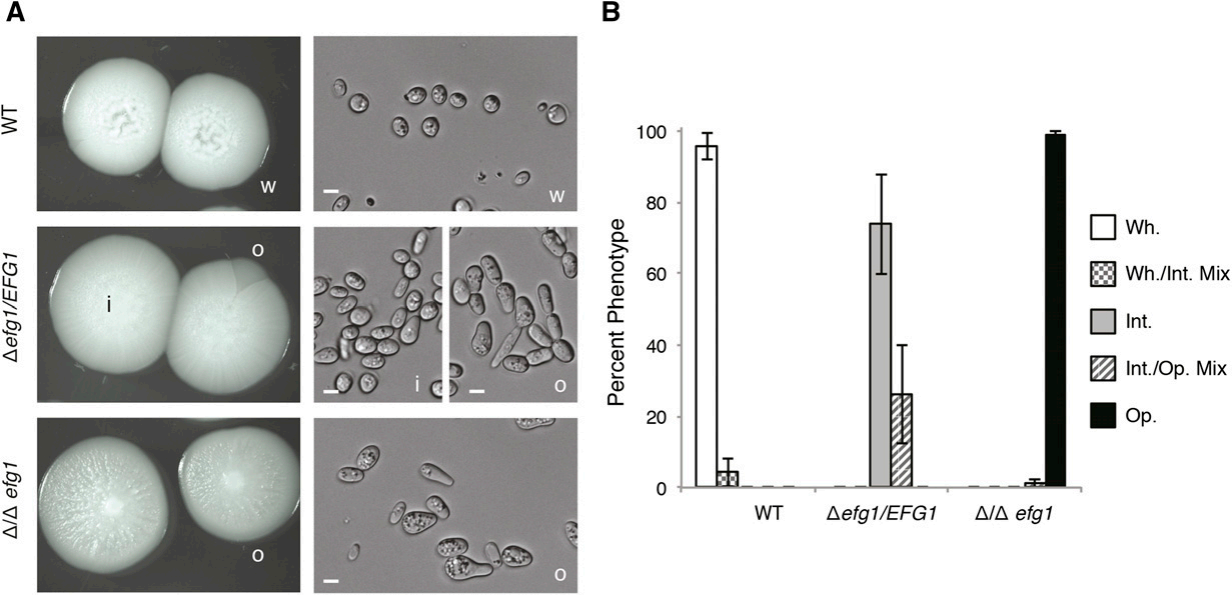
*EFG1* is essential to maintain the white state in *C. tropicalis*. (A) Photos of representative colony and cellular morphologies of the default states of the wild-type, heterozygous *efg1Δ/EFG1*, and homozygous *efg1Δ/efg1Δ* strains in *C. tropicalis* MYA3404 genetic background. Scale bars are 5 μm. w, white; i, intermediate; and o, opaque cell types are indicated. (B) Phenotype frequencies of the same strains starting from a population of white, intermediate, or opaque cells. Cells in these states were used because these were the default states for wild-type, *efg1Δ/EFG1*, and *efg1Δ/efg1Δ* cells, respectively. Error bars are the SD of five independent experiments.

To test whether *EFG1* plays a role in *C. tropicalis* white-opaque switching, we plated single colonies on Spider medium and grew them at 30° for 7 days. Phenotypic switching in *C. tropicalis* involves transitions between white, opaque, and a third phenotypic state that is intermediate to both the white and opaque forms (A. M. Porman, M. Anderson, N. Wang, E. Mancera, and R.J. Bennett, unpublished data). As shown in Figure 3, deletion of *EFG1* had a major effect on the phenotypic states in *C. tropicalis*. The white state was stable in the wild-type background, but heterozygous *efg1Δ/EFG1* and homozygous *efg1Δ/efg1Δ* strains, respectively, formed the "intermediate" or the opaque state as the default state. In terms of switching frequencies, white isolates from wild-type strains showed a low percentage of switching events, with only 4% of colonies showing sectoring to the "intermediate" phenotypic state. In contrast, isolates from heterozygous or homozygous strains showed only switching between the intermediate and opaque states (Figure 3B). These experiments establish that the role of *EFG1* in *C. tropicalis* is similar to that of its ortholog in *C. albicans*, where it acts to promote stable formation of the white state (Srikantha *et al.* 2000; Zordan *et al.* 2007). We also generated *efg1Δ/efg1Δ* strains in a *C. tropicalis* genetic background different from the background used for the genome sequencing project and observed qualitatively the same phenotypes in biofilm formation and white-opaque switching (data not shown).

In summary, the phenotypes of the *C. tropicalis EFG1* deletion mutants establish that this gene plays a key role in filamentation, biofilm formation, and white-opaque switching. These findings are consistent with a conserved role for *EFG1* in regulating morphological transitions in both *C. tropicalis* and *C. albicans*.

## Discussion

The absence of an ortholog of *EFG1* in the assembled genome of *C. tropicalis* was surprising given the conservation of this gene among other CTG species and its importance for regulating several key morphological transitions in *C. albicans*. Here, we report that *C. tropicalis* does indeed contain a true *EFG1* ortholog. The *C. tropicalis* gene is located on the homologous chromosome to the *C. albicans EFG1* gene (chromosome R), but it occupies a different position on this chromosome (Figure 1). Despite its different genomic location, the gene sequence reveals a shared evolutionary history with *EFG1* genes from other species. It is difficult to recreate the exact genomic rearrangements that led to repositioning of *EFG1*, but the fact that the *C. albicans* and *C. tropicalis EFG1* orthologs are on the same chromosome is consistent with the idea that most genomic rearrangements in the CTG clade are intrachromosomal (Butler *et al.* 2009).

We demonstrate, through gene knockouts, that *C. tropicalis EFG1* regulates filamentation, biofilm formation, and white-opaque switching, indicating functional conservation with its ortholog in *C. albicans*. In contrast, analysis of the *EFG1* paralog, *EFH1*, revealed a relatively minor role, if any, in each of these processes in both *C. tropicalis* (this work) and *C. albicans* (Doedt *et al.* 2004). *EFG1* also regulates biofilm formation and a phenotypic switch in *C. parapsilosis*, although the latter appears distinct from that of the white-opaque switch (Connolly *et al.* 2013; Holland *et al.* 2014). It is therefore evident that this master regulator of morphological transitions has a conserved function between several of the most pathogenic *Candida* species. The molecular mechanisms underlying filamentation, biofilm formation, and white-opaque switching have been extensively studied in *C. albicans*, but little is known about these mechanisms in *C. tropicalis*. Identification of the ancient transcription factor *EFG1* in *C. tropicalis* is a key step forward in understanding the strategies used by this species to adapt to the human host.

To gain insight into why the *C. tropicalis EFG1* gene was not present in the published version of the genomic sequence (Butler *et al.* 2009), we examined the assembly process used for *C. tropicalis*. Initially, the genomic sequence was assembled using Arachne with standard parameters (Jaffe *et al.* 2003; Butler *et al.* 2009). However, it was found that many of the genes produced by this assembly were interrupted with stop codons caused by collapsing both haplotypes into a consensus haploid sequence. To address this problem, a new assembly was generated targeting highly polymorphic regions of the genome for iterative reassembly to minimize disagreement in the consensus and phase through a single haplotype for a given region (Butler *et al.* 2009). This strategy solved the problem of stop codon interruptions but reduced the total size of the assembly (8 kb smaller) and created new breaks in three scaffolds. Thus, assembling the *C. tropicalis* genome was a compromise between completeness, contiguity, and accuracy, made particularly challenging by the high number of allelic polymorphisms in the genome. In retrospect, we found that *EFG1* was present in the initial assembly (where many genes were interrupted by stop codons), but was lost in the subsequent assembly that was published. Overall, our results stress the fact (often overlooked by investigators, including us) that published genome sequences are drafts in various stages of completeness. As was the case with the *C. albicans* genome sequence, further curation by the scientific community is critical to complement the computational assembly of genome sequences. This is an especially important and timely reminder in light of the exponential generation of new genomic sequences.
